# Pure Drugs and Medicines

**Published:** 1851-09

**Authors:** 


### ARTICLE II.

### PURE DRUGS AND MEDICINES.

We would call attention to the circular of Philip Schieffelin, Haines & Co. accompanying the present number of the Journal.

The subject of pure and unsophisticated medicines is one of as much importance to the practitioner as any to which his attention can be directed. We have recently, as a member of a committee of the American Medical Association, had occasion to examine into the subject in this region. We are sorry our observations were not received in time to be read at the late meeting of that body. Our information goes to prove that there are many articles of inferior quality, some entirely inert, and others pernicious in their properties, used by the physicians of the North-West. In fact, we learned from reliable sources, that when the pure articles were presented at a higher price than was placed upon the indifferent and sophisticated, many physicians would prefer the latter!!

Certainly in any light in which such a course can be viewed it is miserable policy. If the physician desires the specific effect of a remedy, it is necessary that he should have the genuine article to secure it, without which he will be unable to determine whether his failure, when it occurs, is in consequence of the worthlessness of the article or the impropriety of the prescription. And again, if he wants his articles to be *cheap*, he can buy the pure and adulterate for himself at a greater profit, and enjoy the advantage of knowing of what the adulteration consists.

